# Anti-CCL2: building a reservoir or opening the floodgates to metastasis?

**DOI:** 10.1186/s13058-015-0573-4

**Published:** 2015-05-21

**Authors:** Jessica R Hitchcock, Christine J Watson

**Affiliations:** Department of Pathology, University of Cambridge, Tennis Court Road, Cambridge, CB2 1QP UK

## Abstract

Neutralisation of macrophage chemoattractant C-C chemokine ligand 2 (CCL2) has shown reduced metastasis and enhanced survival in numerous experimental models of tumorigenesis. However, important new findings reported in *Nature* by Momo Bentires-Alj’s laboratory demonstrate that withdrawal of anti-CCL2 treatment accelerates lung metastasis and death in mice. The study highlights the need to consider longer term consequences of therapeutic intervention of metastatic disease, especially with regard to transient interference with the tumour microenvironment.

## Background

The association between macrophage accumulation in primary tumours and poor prognosis is well established, and it is widely acknowledged that macrophages can contribute to metastatic onset [1]. In breast cancer patients, the majority of deaths are associated with metastasis - understanding the relationship between macrophages and metastatic potential will therefore be key to future strategies of therapeutic management. One promising intervention is neutralisation of macrophage attractant C-C chemokine ligand 2 (CCL2), which is thought to target monocyte migration into the tumour microenvironment [2]. Indeed CCL2 depletion by antibody treatment and genetic modification results in reduced metastasis and improved survival in multiple experimental models of mammary cancer [2,3]. Furthermore, elevated CCL2 in breast cancer patients is associated with macrophage infiltration and decreased patient survival [4]. Whilst depletion of CCL2 does not affect primary tumour growth, it is believed that monocytes facilitate metastatic seeding by vascular endothelial growth factor (VEFG)-A-mediated angiogenesis and that this occurs in a CCL2-dependent manner [2]. However, a recent study by Bonapace and colleagues identifies a paradoxical enhancement of disease severity upon withdrawal of anti-CCL2 treatment [5].

## The article

Bonapace and colleagues’ article demonstrates mammary tumour secretion of CCL2, enabling recruitment of inflammatory monocytes to the primary tumour and the metastatic site [5]. The authors build upon the previous observation that CCL2-neutralisation inhibits metastasis [2], demonstrating sequestration of inflammatory monocytes in the bone marrow during treatment. However, the main finding of the study is that interruption of anti-CCL2 treatment results in enhanced metastasis and accelerated death, caused primarily by monocyte release from the bone marrow. The authors demonstrate that monocyte accumulation in the primary tumour results in enhanced blood vessel formation and increased cancer cell mobilisation. Monocyte infiltration of the metastatic site causes increased proliferation of metastatic cells, fuelled by increased production of IL6, and subsequent STAT3 phosphorylation. Finally, angiogenesis in the metastatic site is enhanced by monocyte production of VEGF-A. This study emphasises the tumour microenvironment as the critical determinant of successful anti-metastatic therapy, heeding that extreme caution be exercised when considering anti-CCL2 treatment in metastatic disease.

## Viewpoint

Bonapace and colleagues’ study identifies the surprising consequences of termination of an immunotherapy that has previously shown promise both in the laboratory and in clinical application [2,6-8]. The authors elegantly utilise a series of neutralisation treatments to mechanistically dissect the molecular pathway responsible for the augmented metastatic phenotype described upon cessation of anti-CCL2 therapy. Yet despite this mechanistic understanding, many aspects of the story, particularly in immunological terms, remain unclear.

The immune system must have the ability to detect foreign antigen and respond in an appropriate and timely manner, but must also be adequately desensitized to avoid unnecessary response to self. The majority of studies into CCL2 signalling describe its tumorigenic features, but CCL2 is also known to provide protection, for example, by recruiting anti-tumorigenic T cell subsets to the tumour microenvironment [9]. Therefore, it is plausible that CCL2 signalling may have dual context-dependent effects in tumourigenesis: promoting metastasis of established primary tumours, but performing tumour immune-surveillance in neo-transformed animals [10]. Cancer generally establishes an immune-tolerant environment in which it thrives [1], and this may be key to the observations described by Bonapace et al. Withdrawing CCL2-neutralisation in a tolerogenic environment may favour different outcomes to withdrawing treatment during an immunogenic period.

To determine this hypothesis, further clarification is required to understand the extent to which monocytes are sequestered in the bone marrow during CCL2 neutralisation. It is difficult to interpret the distribution of these cells without appropriate data outlining their localisation and abundance in a resting mouse. This is exacerbated by differing patterns in monocyte distribution between experimental cell lines. Future studies must address bone marrow output during treatment to fully decipher whether monocytes are sequestered here. It is acknowledged that cancer patients often have augmented production of immature cells [11]; therefore some indication as to the functionality of monocytes and their ability to differentiate in the context of CCL2 neutralisation would be useful.

CCL2 neutralisation has shown positive outcomes in several murine models of mammary, prostate and lung cancers [3,12-14]. Despite this, the lack of studies into long-term CCL2 neutralisation and the associated consequences of compromised immunosurveillance, which have been acknowledged in the past [10], are only beginning to be addressed. The work by Bonapace and colleagues has highlighted the necessity of long-term studies, raising important considerations regarding the use of clinical interventions which transiently interfere with the tumour microenvironment (and thus transiently show pleasing therapeutic outcomes). Whether or not the phenotypes observed in implantable cancer models reflect natural onco-metastatic development is unclear. However, the word of caution to future therapeutic strategies becomes increasingly resonant in an era where the expense of immunotherapy plays a prominent role in therapeutic options.

## Abbreviations

CCL2: Chemoattractant C-C chemokine ligand 2
STAT3: Signal transducer and activator of transcription 3
VEFG-A: Vascular endothelial growth factor-A
